# Combined SPRi Sensor for Simultaneous Detection of Nitrate and Ammonium in Wastewater

**DOI:** 10.3390/s21030725

**Published:** 2021-01-21

**Authors:** Martina Vráblová, Ivan Koutník, Kateřina Smutná, Dominika Marková, Nikola Veverková

**Affiliations:** 1 Institute of Environmental Technology, CEET, VSB-Technical University of Ostrava, 17. listopadu 15, 708 00 Ostrava, Czech Republic; ivan.koutnik@vsb.cz (I.K.); katerina.smutna@vsb.cz (K.S.); dominika.markova@vsb.cz (D.M.); nikola.klasova.st@vsb.cz (N.V.); 2 Faculty of Materials Science and Technology, VSB-Technical University of Ostrava, 17. listopadu 15, 708 00 Ostrava, Czech Republic; 3 Faculty of Mining and Geology, VSB-Technical University of Ostrava, 17. listopadu 15, 708 00 Ostrava, Czech Republic

**Keywords:** nitrate, ammonium, surface plasmon resonance, wastewater, aquaponics, sensor

## Abstract

Water pollution is a serious problem in modern society. Agriculture, being responsible for the discharge of agrochemicals, organic matter, or drug residues, produces a huge amount of wastewater. Aquaponics has the potential to reduce both water consumption and the impact of water pollution on fish farming and plant production. In the aquatic environment, inorganic nitrogen is mostly present in the form of nitrate and ammonium ions. Nitrate, as a final product of ammonia mineralization, is the most common chemical contaminant in aquifers around the world. For continuous monitoring of nitrogen compounds in wastewater, we propose a sensor for the simultaneous detection of nitrate and ammonium. A surface plasmon resonance imaging method with enzyme-mediated detection was used. Active layers of nitrate reductase and glutamine synthetase were created on the gold surface of a biochip and tested for the sensing of nitrate and ammonium in water from an aquaponic system. The proposed sensor was applied in water samples with a concentration of NO_3_^−^ and NH_4_^+^ in a range between 24–780 mg·L^−1^ and 0.26–120 mg·L^−1^, respectively, with minimal pretreatment of a sample by its dilution with a buffer prior to contact on a biochip surface.

## 1. Introduction

Water consumption is growing worldwide, whereas its supplies are dwindling. Moreover, water resources all over the world are facing the problem of pollution. Agriculture plays a major role in water pollution [1]. Chemical substances originating from intensively farmed landscape (agrochemicals, organic matter, drug residues, sediments, and saline drainage) reach the groundwater and have a severe impact on water quality [2]. The overuse or misuse of fertilizers in agriculture leads to a large number of nutrients (nitrogen and phosphorus) leaching from soils [3]. An overabundance of nutrients in water associated with the excessive use of fertilizers increases the probability of algae growth, which induces the eutrophication of water bodies [4]. Toxins created by some species of algal blooms can be harmful or even deadly to humans and biodiversity.

In the aquatic environment, inorganic nitrogen is mostly present in the form of nitrate (NO_3_^−^) and ammonium (NH_4_^+^) ions. Ammonium tends to be oxidized to nitrate in the aerobic process of nitrification. Nitrate (NO_3_^−^), as a final product of ammonia mineralization, is the most common chemical contaminant in aquifers around the world [5]. On a global scale, nitrate concentrations may exceed values as high as 110 mg·L^−1^ in surface waters and 440 mg·L^−1^ in groundwater [6]. Elevated nitrate concentration in drinking water poses a serious threat to human health, especially for bottle-fed infants under six months of age, pregnant women, and people with low stomach acid [7,8,9]. The World Health Organization (WHO) thus recommends limiting nitrates in drinking water to 50 mg·L^−1^ [10].

Ammonia (NH_3_) in the environment originates from metabolic, agricultural, and industrial processes, and disinfection with chloramine. Its occurrence in water indicates possible bacterial, sewage, and animal-waste pollution. Natural levels in groundwater and surface water are usually below 0.2 mg·L^−1^, whereas anaerobic groundwater may contain up to 3 mg·L^−1^. Intensive livestock farming can give rise to much higher levels in surface water. The presence of ammonia in an aquatic environment has a negative effect on fish growth, gill condition, organ weights, and hematocrit [11]. Ammonia levels have not been established in drinking water, since it occurs at concentrations well below those of health concern. Toxicological effects for humans are observed only at exposures above about 200 mg/kg body weight [10].

Currently, there is a broad range of analytical methods for nitrate and/or ammonia concentration assessment, including spectrophotometric and fluorometric methods, electrochemical analysis, chromatographic methods, and electrophoretic methods [12,13]. Flow-injection analysis (FIA) techniques can be applied to enhance the efficiency of the analysis. The choice of a suitable method depends on the concentration range and the presence of interferences. Although the chemical reduction of nitrate (NO_3_^−^) to the more reactive nitrite (NO_2_^−^) is often the only way in which the relatively inert nitrate ion can be detected, the use of biocatalytic reduction (using the nitrate reductase enzyme) is possible and offers an advantage over the more toxic chemical-reduction variant, avoiding the use of toxic substances [14,15].

The most widely used methods for the determination of nitrates are spectrophotometric. The most common method is the colorimetric test for the analysis of nitrites and nitrates, for example, the well-known Griess assay [14,16]. In the determination of ammonium, the indophenol blue (IPB) method based on the classic Berthelot reaction is the most widely used spectrophotometric method [17]. Recently, a modified IPB method, replacing toxic and odorous phenol with o-phenylphenol (OPP), was reported [18,19,20,21]. The analyzer developed by Li et al. [19] was used for the online monitoring of ammonium. In the gas-diffusion-based methods, an acid–base indicator (e.g., bromothymol blue, nitrazine yellow) is used, and the color change is measured spectrophotometrically.

On the other hand, the usage of a biological catalyst for electrochemical detection seems to be a more ingenious way to determine nitrate levels, considering the quality of biocatalysts in improving the sensitivity and selectivity of an electrode. In recent years, nitrate biosensors with nitrate reductase as the biological recognition element have gained particular interest as they enable online and continuous monitoring and are nontoxic [22,23,24,25].

The surface plasmon resonance (SPR) of metallic structures permits the identification of pollutant molecules in the environment. A simple colorimetric method for nitrate detection using gold nanorods (AuNRs) was reported by Akbari et al. [26]. Miao et al. [27] have come up with an SPR-based nitrite nanosensor combining surface-modified gold nanoparticles (AuNPs) and the traditional colorimetric detection with a detection limit of 3.0 µg.L^−1^. The use of AuNPs functionalized with Griess reaction reagents was also reported by Daniel et al. [28]. The advantages of a simple and compact probe design, low cost, and suitability for in situ and in vivo measurements are offered by fiber-optic surface plasmon resonance (FOSPR) sensors [29]. A FOSPR-based probe utilizing a nanocomposite of carbon nanotubes/Cu nanoparticles was proposed for the ultratrace sensing of nitrate by Parveen et al. [30]. Moreover, an FOSPR-based sensor proposed by Zhang et al. [31] enables the simultaneous measurement of nitrate concentration and temperature. For the continuous monitoring of nitrate in an aquatic environment, many in situ sensors have been reported [32,33]. However, these technologies have not been adopted on a large scale due to prohibitive costs [34]. Thus, simple low-cost optical detectors are being developed, such as a sensor employing a UV LED source recently designed by [35] or the above-mentioned SPR sensors.

The continuous monitoring of nitrogen nutrients is of particular interest, for example, in aquaponic systems. Aquaponics is a combination of aquaculture and hydroponics, wherein aquaculture is defined as the farming of aquatic organisms including fish, mollusks, crustaceans, and aquatic plants [36], and hydroponics is defined as the production of plants in a soilless medium whereby all of the nutrients supplied to the crop are dissolved in water [37]. In aquaponics, the majority of nutrients required for plant growth in the hydroponic system arise from waste originating from aquaculture [38,39]. The aquaculture effluent contains ammonia, which is transformed to nitrate via nitrifying bacteria. Dissolved nitrate is further exploited as a nutrient source for plants in the hydroponic part. The water remediated of cumulated nutrients is then recycled back to the aquaculture tank [40]. For the smooth operation of aquaponics, it is necessary to keep the concentrations of all nitrogenous compounds within acceptable limits and to avoid an undesirable accumulation of nitrogenous substances in the system [41,42]. Average values in aquaponics come to 60 mg·L^−1^ for nitrate and 15 mg·L^−1^ for total ammonia nitrogen [43].

The aim of this work was to design and test a sensor based on the enzyme-mediated detection of nitrates and ammonium by surface plasmon resonance imaging (SPRi). SPRi with a charge-coupled device (CCD) detector combines a fast detection with a spatial resolution on a biochip surface. Therefore, several enzymes can be simultaneously immobilized on the biochip surface and used for the online detection of two or more compounds dissolved in water. Our research was focused on the detection of nitrate and ammonium ions present in wastewater from aquaponics due to the need for the continuous monitoring of water quality in these systems.

## 2. Materials and Methods

### 2.1. Water Samples

A bare SPRi-Biochip™ with a gold surface (HORIBA France SAS, Longjumeau, France) was tested for sodium, potassium, calcium nitrate, and nitric acid dissolved in water. Sensors (biochips with enzymes immobilized on their surfaces) were tested on model water samples prepared from ammonium sulfate or potassium nitrate dissolved in a mobile phase (Tris-HCl or PBS buffer, respectively). Knop’s solution was prepared by dissolving 1.44 g Ca(NO_3_)·4H_2_O, 0.25 g KH_2_PO_4_, 0.125 g KCl, 0.51 g MgSO_4_·7H_2_O, and 2.0 g FeCl_3_·6H_2_O in ultrapure water. All used chemicals were of p.a. quality. Samples of wastewater were collected from (i) the hydroponic part of a small-scale aquaponic system (50 L), (ii) the aquaculture part of a small-scale aquaponic system (50 L), and (iii) hydroponics that was not connected with aquaculture. The aquaponic system was populated by crucian carps (*Carassius carassius*) and Mexican mint (*Coleus amboinicus*).

### 2.2. Surface Plasmon Resonance Imaging (SPRi) and Biochips Preparation

SPRi detection is based on changes in surface properties due to the interaction of an analyte with an enzyme bound to the gold biochip surface. Model water samples were tested on SPRi-Biochips™ (HORIBA France SAS, Longjumeau, France) with a monolayer of an enzyme. For the detection of nitrate and ammonium, monolayers of nitrate reductase (NR) and glutamine synthetase (GS), respectively, were prepared. Real water samples were tested on a combined biochip with spots of both NR and GS. The principle of enzymatic detection was based on enzymatic assays (Sigma-Aldrich, St. Louis, MO, USA):

$$\mathrm{Nitrate}+\beta \text{-}\mathrm{NADH}\;\overset{\mathrm{Nitrate}\;\mathrm{Reductase}}{\to }\mathrm{Nitrite}+\beta \text{-}\mathrm{NAD}+H_{2}O$$

$$\mathrm{Glutamate}+{\mathrm{NH}}_{4}^{+}+\mathrm{ATP}\;\overset{\mathrm{Glutamine}\;\mathrm{Synthetase}}{\to }L\text{-}\mathrm{Glutamine}+\mathrm{ADP}+\mathrm{Pi}$$

where β-NADH and glutamate are cofactors (chemical compounds that are required for an enzyme’s activity as a catalyst).

#### 2.2.1. Biochips with Monolayers of Nitrate Reductase or Glutamine Synthetase

Nitrate reductase from *Aspergillus niger* (Sigma-Aldrich, St. Louis, MO, USA, CAS 9029-27-0) was dissolved in PBS buffer (10 mM, pH 7.3) in a concentration of 11.2 mg·mL^−1^. L-glutamine synthetase from *Escherichia coli* (Sigma-Aldrich, St. Louis, MO, USA, CAS 9023-70-5) was dissolved in Tris-HCl buffer (20 mM, pH 7.1) in a concentration of 0.672 mg·mL^−1^. Each solution was applied to the surface of one bare SPRi-Biochip™ (with a golden layer, HORIBA France SAS, Longjumeau, France) by a sequence of injections into the measuring cell. The mobile phase in the monolayer application and during experiments was the same buffer used for enzyme dissolution. The contact time between the enzyme and gold surface was 30 min for each injection, and the injections were repeated until the signal stabilized. The effect of binding between the ligand (enzyme) and the analyte (ion) in presence of a cofactor (a catalyst) was detected. As a cofactor, 0.2 mM solution of β-NADH (Sigma-Aldrich, St. Louis, MO, USA) in PBS buffer for experiments with nitrate reductase and 2 mM solution of L-glutamic acid (Sigma-Aldrich, St. Louis, MO, USA) in Tris-HCl buffer for experiments with glutamine synthetase was used.

#### 2.2.2. Combined Biochip with Spots of Nitrate Reductase and Glutamine Synthetase

Solutions of NR and GS in PBS buffer (20 mM, pH 7.2, enzyme concentration of 1 mg·mL^−1^) were put in drops on the surface of CS-LD SPRi-Biochip™ (biochip with a chemically modified gold surface, HORIBA France SAS, Longjumeau, France). Contact time was 2 h. Further, the biochip was rinsed with ultrapure water, blocked by ethanolamine solution (pH 9) for 15 min, and rinsed with ultrapure water again. As a mobile phase, PBS buffer (20 mM, pH 7.2) with dissolved cofactors (0.2 mM β-NADH and 2 mM L-glutamic acid) was used.

#### 2.2.3. Surface Plasmon Resonance Imaging (SPRi)

The measurement was performed on an OpenPlex SPRi instrument (HORIBA France SAS, Longjumeau, France). Optical excitation of surface plasmons was achieved by the method of attenuated total reflection (prism coupling). The measurements were performed at a fixed angle, and the amplitude was measured. The mobile phase (buffer) was degassed through a vacuum degasser and pumped into the apparatus with a constant flow (50 µL.min^−1^) via a peristaltic pump. The measurement of the prepared samples was performed by injecting the analyte through the flow loop (volume 200 μL). The samples were undiluted (model water samples) or diluted ten times by mobile phase (samples from hydroponics and aquaponics, Knop’s solution). Repeatability was calculated during validation of the method from repeated measurements of real samples; the average standard deviation was 1.4% of the mean when the maximum signal of the sensor was read, and 6.9% of the mean when a slope of the affinity curves was calculated.

### 2.3. Ion Chromatography

The nitrate content of samples was determined by measurement on an Eco IC Ion chromatograph with a conductivity detector (Metrohm AG, Herisau, Switzerland). A Metrosep A supp 17 (150/4.0) column and a mobile phase (sodium bicarbonate: 0.2 mmol.L^−1^; sodium carbonate: 5.0 mmol.L^−1^) were used. A mixed standard Astasol (Analytika, Prague, Czech Republic) was used for calibration. Results (expressed in mg·L^−1^) were calculated using MagIC Net 3.2 software (Metrohm AG, Herisau, Switzerland).

### 2.4. UV-VIS Spectrophotometry

The ammonium content of samples was determined by Standard test NANOCOLOR Ammonium (Ammonium-Indophenol method; for concentration range 0.1–2.5 mg∙L^−1^) (Macherey-Nagel GmbH & Co, Düren, Germany). Measurements were performed using a Specord 250 Plus UV-VIS spectrophotometer (Jena Analytik AG, Jena, Germany). Signals were detected in a wavelength of 690 nm.

## 3. Results and Discussion

**Figure 1:**
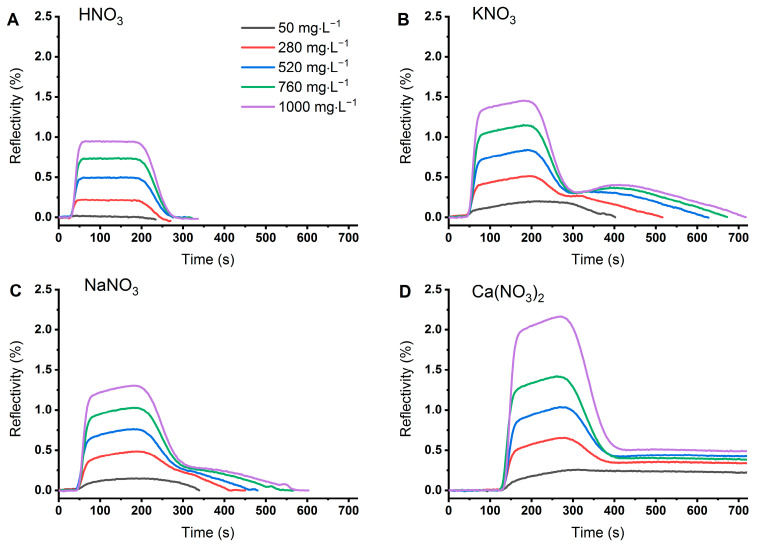
Surface plasmon resonance imaging (SPRi) signals of different forms of nitrates (HNO_3_ (**A**), KNO_3_ (**B**), NaNO_3_ (**C**), Ca(NO_3_)_2_ (**D**)) in water sensed by a biochip with unmodified gold surface.

Experiments based on sensing nitrates in model water samples on the biochip with bare gold surface revealed different peak shapes and areas depending on the cation present in the calibration solution (Figure 1). There was no specific interaction between the gold surface and NO_3_^−^ when HNO_3_ was dissolved in ultrapure water. In that case, the signal was dependent on the concentration of the solution due to the different refractive indices. On the other hand, K^+^, Na^+^, and Ca^2+^ ions interacted with the gold and caused tailing of the peak. Therefore, the sensed signal did not correlate with the concentration of NO_3_^−^ ions and was affected by the type of cation.

**Figure 2:**
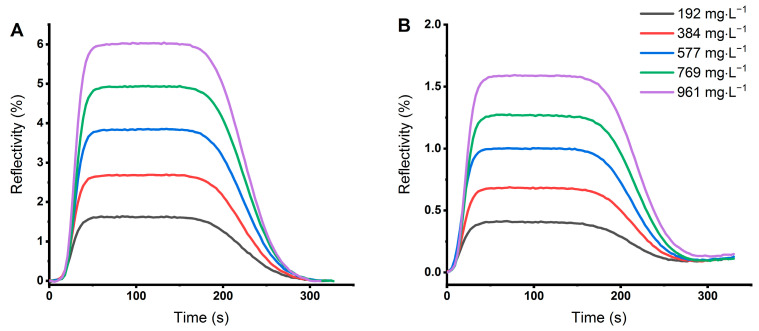
(**A**) SPRi signals of KNO_3_ dissolved in PBS buffer (pH 7.3) sensed on a monolayer of nitrate reductase and (**B**) SPRi signal of (NH₄)₂SO₄ dissolved in Tris buffer (pH 7.1) sensed on a monolayer of glutamine synthetase.

A monolayer of the enzyme nitrate reductase (NR) on the gold surface prevented the nonspecific interaction between an analyte and the sensor surface. In this case, NR was a specific ligand for the analyte (NO_3_^−^) dissolved in an aqueous sample [24,44]. Experiments with KNO_3_ dissolved in PBS buffer proved a good correlation between the concentration of NO_3_^−^ ions and the height of the peak (Figure 2).

**Figure 3:**
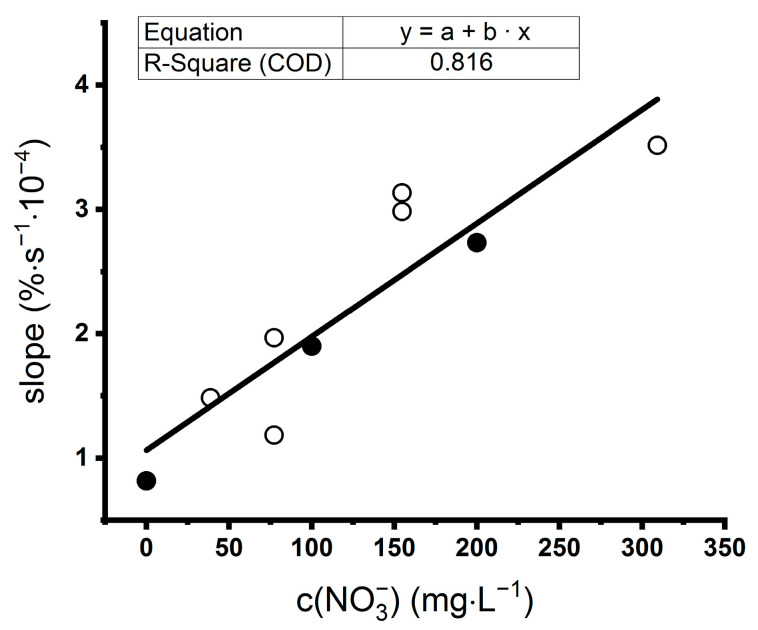
Detection of nitrate in Knop’s solution (white circles) used in hydroponics and a standard solution of KNO_3_ (black circles) on a monolayer of nitrate reductase (NR). KNO_3_ was dissolved in PBS buffer (pH 7.3), and the Knop’s solution was diluted by this PBS buffer ten times. A slope of the SPRi signal reflecting the interaction between nitrate and NR was calculated from recorded peaks of reflectivity. Linear regression was calculated from all points.

A similar system of specific ligand and analyte can be found for ammonium ions dissolved in Tris buffer, where sensing on a monolayer of the enzyme glutamine synthetase (GS) led to symmetric peaks with a height dependent on the concentration of NH_4_^+^ ions (Figure 2B). Buffers were used with respect to the chemical properties of the enzymes [45,46], and ensured a stable pH because pH can influence the enzymatic activity and thus the SPR signal [44,47]. In methods based on surface plasmon resonance, the signal depends not only on the specific interaction between an analyte and a ligand but also on the refractive index of the solution [48]. Therefore, the height or area of the peak can only be used as a concentration indicator for one-component solutions. In real samples, a mixture of compounds is present and matrix effects appear [49]. To solve this problem, the slope of the signal in a period of peak “plateau” (e.g., 50–150 min in our case) can be evaluated. To verify this approach, we measured samples of Knop’s solution and a standard solution of KNO_3_ diluted by PBS buffer to obtain different concentrations of NO_3_^−^ and found a good correlation between the slope of the peak and the concentration of NO_3_^−^ ions (Figure 3).

**Figure 4:**
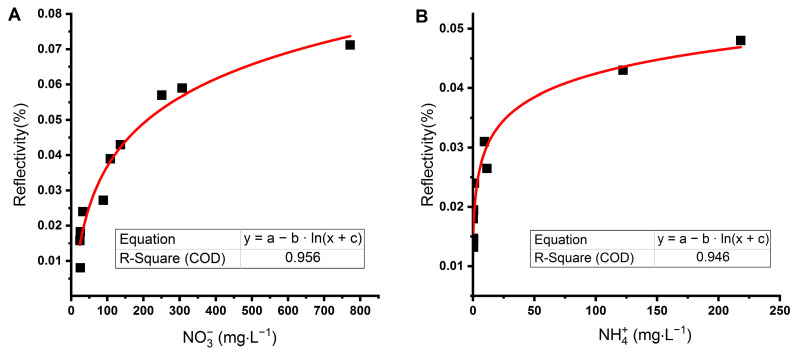
The relationship between SPRi signals measured by combined biosensors for the simultaneous detection of nitrate (**A**) and ammonia (**B**) and the concentration of ions in water samples from aquaponics measured by conventional methods. The sensor surface was modified by nitrate reductase (NO_3_^−^ detection) and glutamine synthetase (NH_4_^+^ detection). The signal from the unmodified part of the sensor was used as a reference.

Another approach is to use a two-channel SPR [50]. This was not possible in our case due to the one-channel construction of our system. Nevertheless, the SPR imaging allows using different areas on the biochip surface to subtract signals acquired by specific and nonspecific interactions. Therefore, we prepared a biochip with three measuring zones: enzymes (NR and GS) formed two zones, and the third zone was a reference formed by a blocked surface of the CS-LD biochip that could not interact with ions of our interest (NO_3_^−^ and NH_4_^+^). SPR imaging allowed scanning of the whole area of the measuring zone of the biochip at one time due to the CCD detector [51,52]. We sensed the signal from enzymes and subtracted the signal from the reference area. This led to the reduction of matrix effects. Then, we recorded the maximum relative reflectivity for each sample obtained on both NR and GS surfaces. The detection limit of the sensor was 17.8 mg·L^−1^ for nitrate and 0.115 mg·L^−1^ for ammonium; therefore, only samples with a higher concentration of ions were evaluated. Selected water samples from aquaponics and hydroponics had concentrations of NO_3_^−^ and NH_4_^+^ between 24 and 773 mg·L^−1^ and 0.26 and 219 mg·L^−1^, respectively (Table 1). Samples were diluted ten times in a buffer to control pH and to lower matrix effects. Individual measurements on enzymes revealed a nonlinear relationship between concentration measured by an independent method (ion chromatography or spectrophotometry) and the signal from the sensor (Figure 4).

**Figure 5:**
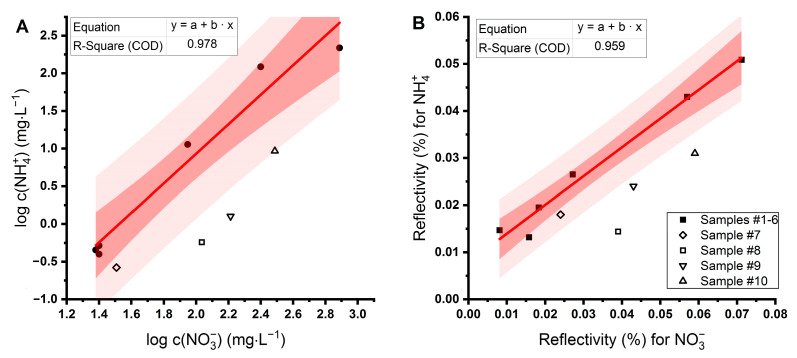
(**A**) The relationship between concentrations of nitrate and ammonium in samples from aquaponics and hydroponics measured by ion chromatography (nitrate) and UV-VIS spectrophotometry (ammonium). (**B**) Signals from the SPRi sensor for the simultaneous detection of nitrate and ammonium. A series of six samples were used for linear fit (closed symbols), and another four samples for validation of the sensor (open symbols). Samples that are out of the prediction band or higher than the set limit (which is optional and corresponds to the maximum required concentration) may predict an unwanted state of nutrients in water.

Six samples (1–6) were used to test the sensor, and another four samples (7–10) were used to verify results. We plotted ion concentrations (Figure 5) obtained from ion chromatography (NO_3_^−^) and spectrophotometry (NH_4_^+^) on a logarithmic scale. The first group of samples was fitted with the linear function, and the confidence and prediction intervals were calculated. From the second group of samples (Figure 5A, open symbols), three were outside the prediction interval with a relatively higher concentration of nitrate than ammonium ions compared to the rest of the samples (8–10).

The next step was to measure the same ten samples on the combined biochip by SPRi and correlate the reflectivity sensed on NR (belonging to NO_3_^−^ ions) and GS (belonging to NH_4_^+^ ions) (Figure 5B). When applying the same protocol for data fitting, the outliers were identified as samples 8–10. The agreement between the relationship of concentrations measured by convenient methods and signals from the sensor suggests the possibility of using the sensor to detect deviations in nitrogen-compound concentrations when monitoring the state of the aquaponic system. The simultaneous assessment of nitrate and ammonia allows evaluation of the balance between aquaculture (a donor of nitrogen) and hydroponics (an acceptor of nitrogen) as well as the proper function of nitrifying bacteria.

The advantages and disadvantages of SPR-based sensors were discussed by Piliarik et al. [53]. We proposed a sensor based on SPR imaging with amplitude measurement. SPRi-based sensors are often used for the detection of small molecules [54,55,56,57] and the detection of pollutants in water [58,59,60]. An application of SPRi in spatially differentiated sensing (“electronic tongue”) was reported by Genua et al. [61].

In aquaponics and hydroponics, concentrations of nitrogen compounds in water are relatively high in order to ensure the rapid growth of plants. Ru et al. [62] reached a limit of up to 35 mg·L^−1^ of ammonia in the aquaponic system. Generally, nitrate is a nontoxic compound which can be found at levels exceeding 1000 mg·L^−1^ in freshwater environments without adverse effects on aquatic organisms [63]. In aquaponic systems, nitrate has been reported to be harmless at concentrations of 150–300 mg·L^−1^ [41,64]. Therefore, the low detection limit for nitrates and ammonium is not a key factor for used sensors. The main task is to sense changes in the balance of nutrients in a timely manner. Sensors for monitoring water quality should be user-friendly, fast, and fully automated, similar to sensors for water temperature, water flow rate, light intensity, pH level, and plant height [65,66]. Moreover, the simultaneous measurement of multiple parameters of water quality is advantageous to lower operational costs [67]. Therefore, the SPRi sensor proposed in this work could be successfully used in aquaculture and hydroponic or aquaponic systems to improve the monitoring of water quality, thus increasing the efficiency of fish and plant production while reducing the amount of discharged wastewater.

## 4. Conclusions

A combined sensor for the simultaneous detection of nitrate and ammonium in water was proposed to test the biosensor technology as an alternative to the chemical detection methods for nitrogen-compound determination. Surface plasmon resonance imaging with the enzymes nitrate reductase and glutamine synthetase immobilized on the surface of a biochip was used as a detection method. The proposed sensor was tested for standard solutions of nitrate and ammonium as well as real wastewater samples from aquaponics. The advantage of the SPRi-based sensor is the ability to be used for online and semicontinuous monitoring of several compounds at the same time with only minimal sample pretreatment.

## Figures and Tables

**Table 1:** Concentrations of nitrate and ammonium in water samples from aquaponics and hydroponics measured by ion chromatography (NO_3_^−^) and UV-VIS spectrophotometry (NH_4_^+^).

Sample No.	c (NO_3_^−^) mg·L^−1^	c (NH_4_^+^) mg·L^−1^
1	24.0	0.45
2	25.1	0.40
3	25.2	0.52
4	88.7	11.34
5	251.0	122.29
6	772.4	218.14
7	32.2	0.26
8	108.3	0.57
9	136.3	1.27
10	307.0	9.31

## Data Availability

The data presented in this study are available on request from the corresponding author.
